# Burden of injuries in Nepal, 1990–2017: findings from the Global Burden of Disease Study 2017

**DOI:** 10.1136/injuryprev-2019-043309

**Published:** 2020-01-08

**Authors:** Puspa Raj Pant, Amrit Banstola, Santosh Bhatta, Julie A Mytton, Dilaram Acharya, Suraj Bhattarai, Catherine Bisignano, Chris D Castle, Govinda Prasad Dhungana, Zachary V Dingels, Jack T Fox, Pawan Kumar Hamal, Zichen Liu, Narayan Bahadur Mahotra, Deepak Paudel, Khem Narayan Pokhrel, Chhabi Lal Ranabhat, Nicholas L S Roberts, Dillon O Sylte, Spencer L James

**Affiliations:** 1 Centre for Academic Child Health (CACH), University of the West of England, Bristol, UK; 2 Department of Research, Public Health Perspective Nepal, Pokhara-Lekhnath Metropolitan City, Nepal; 3 Department of Nursing and Midwifery, University of the West of England, Bristol, UK; 4 Department of Preventive Medicine, Dongguk University, Gyeongju, South Korea; 5 Department of Community Medicine, Kathmandu University, Devdaha, Nepal; 6 London School of Hygiene & Tropical Medicine, London, UK; 7 Nepal Academy of Science & Technology, Patan, Nepal; 8 Institute for Health Metrics and Evaluation, University of Washington, Seattle, Washington, USA; 9 Department of Microbiology, Far Western University, Mahendranagar, Nepal; 10 Department of Anaesthesiology and Intensive Care, National Academy of Medical Sciences, Kathmandu, Nepal; 11 Journal of Nepal Health Research Council, Nepal Health Research Council, Kathmandu, Nepal; 12 Institute of Medicine, Tribhuvan University, Kathmandu, Nepal; 13 Health, Nutrition and HIV/AIDS Program, Save the Children, Kathmandu, Nepal; 14 Center for International Health, Ludwig Maximilians University, Munich, Germany; 15 HIV and Mental Health Department, Integrated Development Foundation Nepal, Kathmandu, Nepal; 16 Policy Research Institute, Kathmandu, Nepal; 17 Institute for Poverty Alleviation and International Development, Yonsei University, Wonju, South Korea

**Keywords:** descriptive epidemiology, burden of disease, epidemiology

## Abstract

**Background:**

Nepal is a low-income country undergoing rapid political, economic and social development. To date, there has been little evidence published on the burden of injuries during this period of transition.

**Methods:**

The Global Burden of Disease Study (GBD) is a comprehensive measurement of population health outcomes in terms of morbidity and mortality. We analysed the GBD 2017 estimates for deaths, years of life lost, years lived with disability, incidence and disability-adjusted life years (DALYs) from injuries to ascertain the burden of injuries in Nepal from 1990 to 2017.

**Results:**

There were 16 831 (95% uncertainty interval 13 323 to 20 579) deaths caused by injuries (9.21% of all-cause deaths (7.45% to 11.25%)) in 2017 while the proportion of deaths from injuries was 6.31% in 1990. Overall, the injury-specific age-standardised mortality rate declined from 88.91 (71.54 to 105.31) per 100 000 in 1990 to 70.25 (56.75 to 85.11) per 100 000 in 2017. In 2017, 4.11% (2.47% to 6.10%) of all deaths in Nepal were attributed to transport injuries, 3.54% (2.86% to 4.08%) were attributed to unintentional injuries and 1.55% (1.16% to 1.85%) were attributed to self-harm and interpersonal violence. From 1990 to 2017, road injuries, falls and self-harm all rose in rank for all causes of death.

**Conclusions:**

The increase in injury-related deaths and DALYs in Nepal between 1990 and 2017 indicates the need for further research and prevention interventions. Injuries remain an important public health burden in Nepal with the magnitude and trend of burden varying over time by cause-specific, sex and age group. Findings from this study may be used by the federal, provincial and local governments in Nepal to prioritise injury prevention as a public health agenda and as evidence for country-specific interventions.

## Introduction

About 90% of total deaths and disability secondary to injuries occur in low/middle-income countries (LMIC). Despite economic development in many LMICs, healthcare infrastructure may have limited capacity to cope with this injury burden. In addition, injury burden is a particularly an important topic in areas of the world that have been subject to natural disaster and high rates of conflict that cause large increases in morbidity and mortality from injuries. As with other low-income countries, injury is recognised as an important public health issue in Nepal,^1^ yet no reliable and comprehensive national estimates for injury burden have been published outside of the Global Burden of Disease Study (GBD). Therefore, GBD estimates are a unique source of health information in Nepal.

GBD 2017 produced a comprehensive set of estimates of the injury burden in Nepal. The study estimated death and disability from every cause of injury for all ages and both sexes over a time range spanning from 1990 to 2017.^2–7^ Past research and commentaries have focused on changes in the injury burden in Nepal and have attributed trends in injury burden to rapid urbanisation, infrastructure development, increasing numbers of new and old vehicles (mainly two-wheeler motorised vehicles), poorly designed roads, limited prehospital and emergency care, poor healthcare infrastructure and rehabilitation services, an ageing population and natural disasters.^8–17^ Catastrophic events in Nepal, such as the 2015 massive earthquake and its aftershocks, which killed approximately 9000 and injured over 22 000 people, are known to contribute significantly to the burden of injury.^14^

While many injuries are preventable, Nepal’s national health agenda has not prioritised injury prevention until recently and previous literature has cited a lack of availability of trauma care.^15^ The Nepal Health Sector Strategy (NHSS) 2015–2020,^17^ the NHSS Implementation Plan 2016–2021,^18^ the Nepal Road Safety Action Plan 2013–2020^18^ and the 14th National Plan 2017–2020^19^ have included road safety and postcrash response but have not recognised other types of injury. NHSS 2015–2020 recognised road injuries as Goal 7 and proposed a specific target to reduce life lost due to road injuries to 17 people per 100 000 by 2020.^16 17^ Given the urgency of action to address road injuries, the United Nations General Assembly and its member states adopted a resolution on ‘Improving global road safety’ in 2016, which the government of Nepal endorsed.^20^

Past research has provided estimates of Nepal’s burden of many communicable^21 22^ and non-communicable diseases.^23^ However, research on injuries is limited. The majority of publications reporting injury outcomes are hospital case series with few publications using methodologies that have addressed known sources of bias. Community-based studies have been performed for road injuries,^8^ falls,^24^ fires^25^ and among children,^26^ though not all studies have been conducted in a representative manner. Due to the lack of routinely collected data on injuries and associated risk factors, the burden of injury in Nepal has not been comprehensively measured or discussed in research outside of the GBD study. Thus, it is important to conduct a comprehensive estimate of the burden of injuries in Nepal as well as a contextual review of the burden.

The purpose of this study was to use data from the GBD to explore injury-specific mortality rates for Nepal between 1990 and 2017, disaggregated by sex and age, and to quantify the burden of unintentional and intentional injures in terms of mortality and disability-adjusted life years (DALYs).

## Methods

We analysed results from GBD 2017, which is a collaborative, comprehensive research study conducted by a global network of more than 3150 collaborators, the majority of whom are in LMICs. Methods for GBD 2017 are described in more detail in GBD literature and in online supplementary appendix 1, but in summary, GBD 2017 measured morbidity and mortality from 355 diseases and injuries, 2982 conditions resulting from these diseases and injuries and 89 risk factors or combinations of risks.^1–6^ GBD 2017 used all available data for mortality, population, fertility, cause of death, incidence, prevalence and other epidemiological measures. In order to harmonise heterogeneous data and use all possible sources of data, GBD 2017 applied a broad suite of statistical modelling tools to develop estimates for all outcomes of interest across 195 countries and territories, including Nepal. GBD 2017 generated a complete set of estimates for cause-specific mortality, years of life lost (YLLs), years lived with disability (YLDs) and DALYs from 1990 to 2017.

10.1136/injuryprev-2019-043309.supp1 Supplementary data

In GBD 2017, the data sources used to estimate injury mortality from all countries included vital registration (VR), verbal autopsy (VA), mortality surveillance, census, survey and police record data. Police and crime reports were data sources uniquely used for the estimation of deaths from road traffic injury and interpersonal violence. The police data were collected from published studies, national agencies and institutional surveys such as the United Nations Crime Trends Survey and the WHO Global Status Report on Road Safety Survey. GBD 2017 did not have any available VR, VA or police record data for use in cause of death modelling and estimation, but did use important sources for non-fatal health loss measurement such as national hospital discharge records. A list of injury morbidity and mortality data input sources that were used in GBD 2017 for Nepal is provided in table 1.

**Table 1:** Data sources that were used in GBD 2017 for Nepal injury morbidity and mortality

Purpose	Citation
Causes of death, conflict and disasters	Centre for Research on the Epidemiology of Disasters (CRED). EM-DAT: The OFDA/CRED International Disaster Database. Brussels, Belgium: Catholic University of Leuven.
	Macro International, Inc, Ministry of Health and Population (Nepal), New ERA. Nepal Demographic and Health Survey 2006. Fairfax, United States: ICF International.
	United Nations Office on Drugs and Crime (UNODC). United Nations Surveys on Crime Trends and the Operations of Criminal Justice Systems 1970–2006 as provided by Kavi Bhalla.
	International Institute for Strategic Studies. International Institute for Strategic Studies Armed Conflict Database. London, United Kingdom: International Institute for Strategic Studies.
	Tielsch JM, Khatry SK, Stoltzfus RJ, Katz J, LeClerq SC, Adhikari R, Mullany LC, Black R, Shresta S. Effect of daily zinc supplementation on child mortality in southern Nepal: a community-based, cluster randomised, placebo-controlled trial. Lancet. 2007; 370(9594): 1230–9.
	United Nations Office on Drugs and Crime (UNODC). United Nations Office on Drugs and Crime Global Study on Homicide 2011. Vienna, Austria: United Nations Office on Drugs and Crime (UNODC), 2011.
	Center for Research on Environment Health and Population Activities (Nepal), Ministry of Health (Nepal), New ERA, Options UK, Safe Motherhood Network Federation (Nepal). Nepal Maternal Mortality and Morbidity Study 2008–2009.
	Climate Change and African Political Stability Project (CCAPS). Armed Conflict Location and Event Dataset, Realtime—Robert S. Strauss Center as referenced in Raleigh, Clionadh, Andrew Linke, Havard Hegre and Joakim Karlsen. 2010. Introducing ACLED-Armed Conflict Location and Event Data. Journal of Peace Research 47(5), 651–60.
	Department of Peace and Conflict Research, Uppsala University. UCDP/PRIO Armed Conflict Database Version 4, 2015—UCDP. Uppsala, Sweden: Department of Peace and Conflict Research, Uppsala University, 2014.
	Department of Peace and Conflict Research, Uppsala University. UCDP One-Sided Violence Dataset, Version 1.4, 2015. Uppsala, Sweden: Department of Peace and Conflict Research, Uppsala University, 2015.
	Department of Peace and Conflict Research, Uppsala University. UCDP Battle-Related Deaths Database, Version 5, 2016. Uppsala, Sweden: Department of Peace and Conflict Research, Uppsala University, 2015.
	Department of Peace and Conflict Research, Uppsala University. UCDP Nonstate Conflict Dataset, Version 2.5, 2016. Uppsala, Sweden: Department of Peace and Conflict Research, Uppsala University, 2013.
	Aon Benfield. Global Catastrophe Recap October 2016. London, United Kingdom: Aon Benfield, 2016.
	Department of Peace and Conflict Research, Uppsala University. UCDP Georeferenced Event Dataset, Version 17.1, 2016. Uppsala, Sweden: Department of Peace and Conflict Research, Uppsala University, 2017.
	National Consortium for the Study of Terrorism and Responses to Terrorism (START). University of Maryland. Global Terrorism Database. 2017.
	Pokharel S. Nepal bus crash kills at least 33. Cable News Network (CNN) (Internet). 2016 Aug. 15; World—Asia.
	Pokharel S, Botelho G. Nepal bus crash kills at least 30. Cable News Network (CNN) (Internet). 2015 Nov. 3; World—Asia.
	Nepal bus crash kills 29 people and injures dozens. British Broadcasting Corporation (BBC) (Internet). 2014 Oct. 7; World—Asia.
	Yogacharya KS, Gautam DK. Floods in Nepal: Genesis, magnitude, frequency and consequences. Proc. of the International Conference on Hydrology and Climate Change in Mountainous Areas, November 15–17, 2008, Kathmandu, Nepal. 2008.
	Domonoske C. Floods in South Asia have killed more than 1000 people this summer. National Public Radio (NPR). 2017.
Non-fatal health loss	Centre for Research on the Epidemiology of Disasters (CRED). EM-DAT: The OFDA/CRED International Disaster Database. Brussels, Belgium: Catholic University of Leuven.
	ICF Macro, Ministry of Health and Population (Nepal), New ERA. Nepal Demographic and Health Survey 2011. Fairfax, United States: ICF International.
	World Health Organization (WHO). Nepal World Health Survey 2003. Geneva, Switzerland: World Health Organization (WHO), 2005.
	Lamichhane P, Puri M, Tamang J, Dulal B. Women's status and violence against young married women in rural Nepal. BMC Womens Health. 2011; 19.
	Department of Health Services, Ministry of Health and Population (Nepal). Nepal Hospital Inpatient Discharges 2013–2014.
	Department of Health Services, Ministry of Health and Population (Nepal). Nepal Hospital Inpatient Discharges 2010–2012.

GBD, Global Burden of Disease Study.

For this study, we downloaded GBD 2017 estimates for Nepal from the openly available online resources on the Global Health Data Exchange (GHDx)^27^ and the GBD Results Tool^28^ with additional insights drawn from corresponding data visualisations.^29^ Specifically, we obtained GBD summary results (deaths, DALYs, YLLs and YLDs) for all causes of injury at all levels between 1990 and 2017 via the GHDx GBD Results Tool. To describe the data, we reviewed a list of GBD categorisation of injuries (table 2). We used the GBD 2017 hierarchy of causes that organises injuries into four levels of classification. The highest level of classification in GBD describes disease mortality in three broad categories: communicable, maternal, neonatal and nutritional disorders (group 1); non-communicable diseases (group 2); and injuries (group 3). The number, proportion, ranks, time trend and rates of death and DALYs were presented by different age groups and types of injuries with 95% uncertainty intervals (UI). DALYs data were used to determine rankings of leading causes of injury for all ages and both sexes.

**Table 2:** Categorisation of injuries in GBD 2017

Category of injuries	Cause-specific injuries according to the GBD
A. Transport injuries	
1. Transport injuries	C.1.1 Road injuries: C.1.1.1 Pedestrian road injuries C.1.1.2 Cyclist road injuries C.1.1.3 Motorcyclist road injuries C.1.1.4 Motor vehicle road injuries C.1.1.5 Other road injuries C.1.2 Other transport injuries
B. Unintentional injuries	
1. Falls	C.2.1 Falls
2. Drowning	C.2.2 Drowning
3. Burn	C.2.3 Fire, heat and hot substances
4. Poisonings	C.2.4 Poisonings: C.2.4.1 Poisoning by carbon monoxide C.2.3.2 Poisoning by other means
5. Exposure to mechanical forces	C.2.5 Exposure to mechanical forces: C.2.5.1 Unintentional firearm injuries C.2.5.2 Other exposure to mechanical forces
6. Adverse effects of medical treatment	C.2.6 Adverse effects of medical treatment
7. Animal related	C.2.7 Animal contact: C.2.7.1 Venomous animal contact C.2.7.2 Non-venomous animal contact
8. Foreign body	C.2.8 Foreign body: C.2.8.1 Pulmonary aspiration and foreign body in airway C.2.8.2 Foreign body in eyes C.2.8.3 Foreign body in other body part
9. Environmental heat and cold exposure	C.2.9 Environmental heat and cold exposure
10. Forces of nature	C.2.10 Exposure to forces of nature
11. Other unintentional injuries	C.2.11 Other unintentional injuries
C. Intentional injuries	
1. Self-harm	C.3.1 Self-harm: C.3.1.1 Self-harm by firearm C.3.1.2 Self-harm by other specified means
2. Interpersonal violence	C.3.2 Interpersonal violence: C.3.2.1 Physical violence by firearm C.3.2.2 Physical violence by sharp object C.3.2.3 Sexual violence C.3.2.4 Physical violence by other means
3. Conflict and terrorism	C.3.3 Conflict and terrorism C.3.4 Executions and police conflict

GBD, Global Burden of Disease Study.

The GBD study complies with the Guidelines of Accurate and Transparent Health Estimate Reporting (GATHER) recommendations.^30^ GATHER guidelines are provided in online supplementary appendix 2.

10.1136/injuryprev-2019-043309.supp2 Supplementary data

## Results

Summary results are as follows. Additional results by age, sex, year, and injury cause and nature are available online at www.healthdata.org.

### All injuries

**Figure 1:**
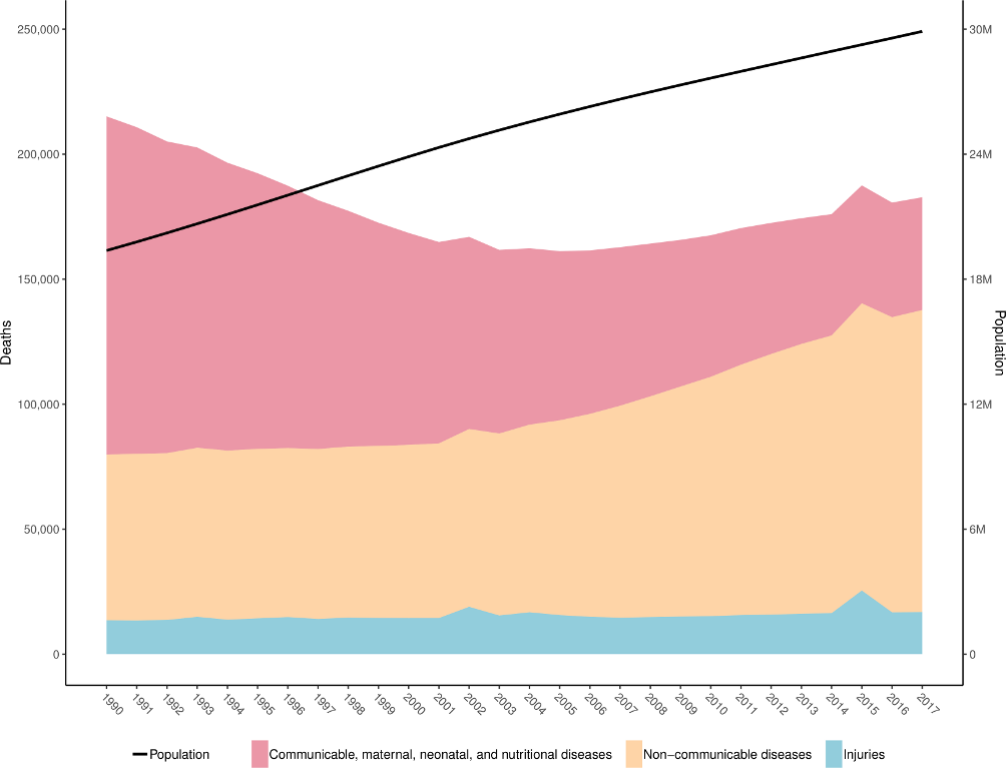
Deaths for both sexes and all ages by level 1 causes and total population, Nepal, 1990–2017.

The trends in numbers of deaths between 1990 and 2017, as well as trends in overall population during this time, are shown in figure 1. During this period, the population of Nepal increased from 19 373 251 to 29 891 524, representing a growth of approximately 2% per year. The figure also shows that despite population growth, the overall number of deaths for all causes steadily declined between 1990 and the early 2000s before gradually increasing up to 2017. By way of comparison, the figure also shows how the number of deaths due to non-communicable diseases has generally increased over time, while the number of deaths due to communicable, maternal, neonatal and nutritional diseases has decreased.

In 2017, there were 16 831 (95% UI 13 323 to 20 579) deaths from injuries in Nepal out of 182 751 (166 242 to 197 584) deaths from all causes (online supplementary appendix table 1). These deaths caused 715 602 (540 423 to 894 816) YLLs and 182 367 (138 390 to 231 736) YLDs. YLLs and YLDs together formed a total of 897 969 (718 350 to 1 092 186) DALYs.

10.1136/injuryprev-2019-043309.supp3 Supplementary data

In terms of percentage of all-cause mortality, in 1990, 6.31% (4.87% to 7.34%) of all deaths were attributed to injuries. By 2017, this number had increased to 9.21% (7.45% to 11.25%) of all deaths. However, the age-standardised mortality rate from injuries decreased from 88.91 (71.54 to 105.31) deaths per 100 000 in 1990 to 70.25 (56.75 to 85.11) deaths per 100 000 between 1990 and 2017, representing a 21% (0.4% to 37.7%) decrease.

### Injury types

**Figure 2:**
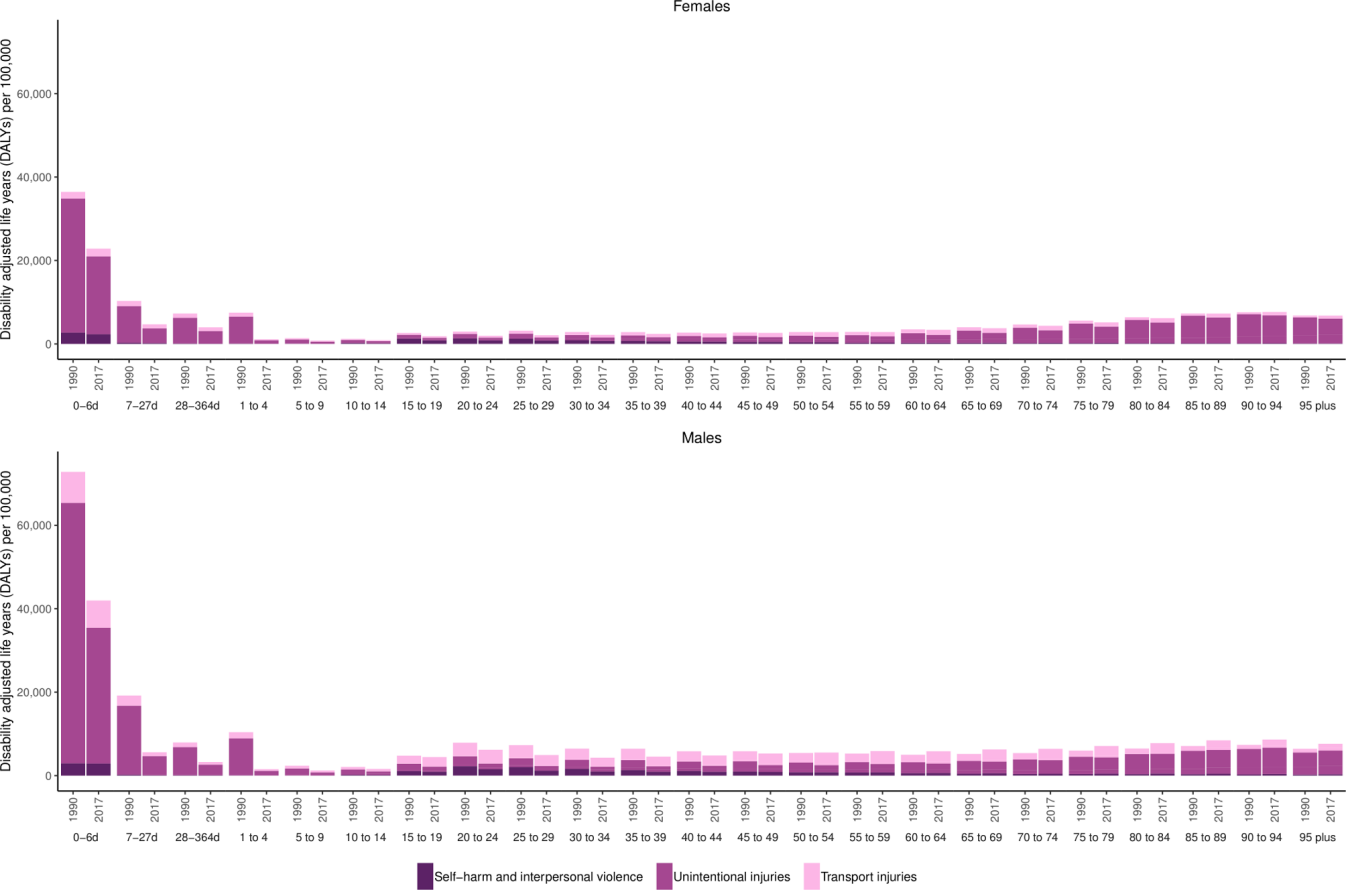
DALYs per 100 000 by age group, sex and year (1990–2017) for females and males for level 2 injury causes in the Global Burden of Disease Study (GBD) cause hierarchy.

The GBD broadly groups injuries into transport injuries, unintentional injuries, and self-harm and interpersonal violence. In 2017, 4.11% (2.47% to 6.10%) of all deaths in Nepal were attributed to transport injuries, 3.54% (2.86% to 4.08%) were attributed to other types of unintentional injuries (including falls 1.59% (1.28% to 1.95%), drowning 0.51% (0.40% to 0.61%), animal related 0.24% (0.1% to 0.36%) and fire/burns 0.20% (0.14% to 0.29%)) and 1.55% (1.16% to 1.85%) were attributed to self-harm and interpersonal violence. The numbers of total DALYs attributed to these three categories by age group and sex for 1990 and for 2017 are shown in figure 2. This figure shows that injury burden in neonatal age groups up to 10–14 years of age as well as older adults aged 55+ years is primarily attributed to unintentional injuries. Conversely, transport injuries and self-harm and interpersonal violence are more important factors of overall injury burden in age groups from 15 to 54 years. The percentage of total DALYs attributed to injuries increased from 5.9% (4.5% to 7.0%) in 1990 to 9.97% (8.1% to 11.9%) in 2017.

### Transport injuries

**Figure 3:**
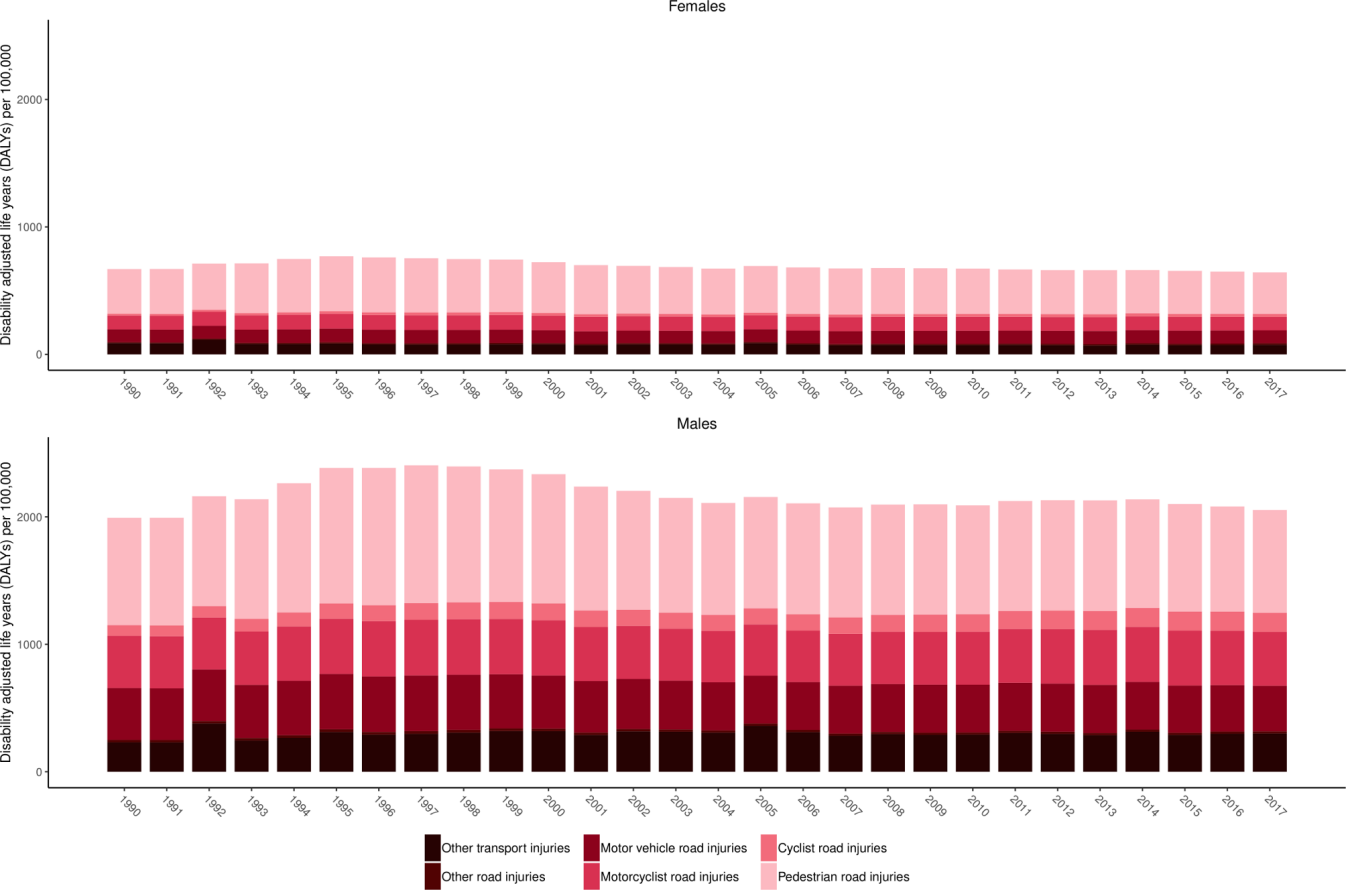
Age-standardised DALYs per 100 000 by year for females and males for level 3 transport injury subcauses in the Global Burden of Disease Study (GBD) cause hierarchy between 1990 and 2017.

Figure 3 shows age-standardised DALYs per 100 000 for transport injuries by subtype of injury between 1990 and 2017 for females and males. This figure shows several trends. First, it demonstrates the higher rate of DALYs from transport injuries in males versus females. Second, it shows that pedestrian road injuries are the predominant type of transport injury DALYs across the time period in both sexes. Third, it shows that the increase in transport injury DALYs during mid-1990s has now started to decline. Associated results are provided in online supplementary appendix table 1. In 1990, there were 3004 (1819 to 4167) deaths due to transport injuries in Nepal causing 164 258 (96 895 to 224 679) YLLs and 12 951 (9289 to 17 004) YLDs forming a total of 177 209 (109 824 to 237 386) DALYs. By 2017, there were 7524 (4480 to 11 214) deaths due to transport injuries in Nepal causing 330 555 (200 266 to 507 611) YLLs and 41 749 (29 999 to 55 428) YLDs forming a total of 372 304 (240 203 to 546 069) DALYs. In Nepal, 90% of all Transport Injuries were 'road injuries' and were the cause of 26.99% (21.35% to 34.71%) of all-injury mortality in 1990 and rose to 40.02% (28.18% to 54.06%) in 2017. In 1990, road injuries ranked 13th in total number of deaths for all ages, whereas in 2017, this injury category ranked 7th in terms of total deaths for all ages.

### Unintentional injuries

**Figure 4:**
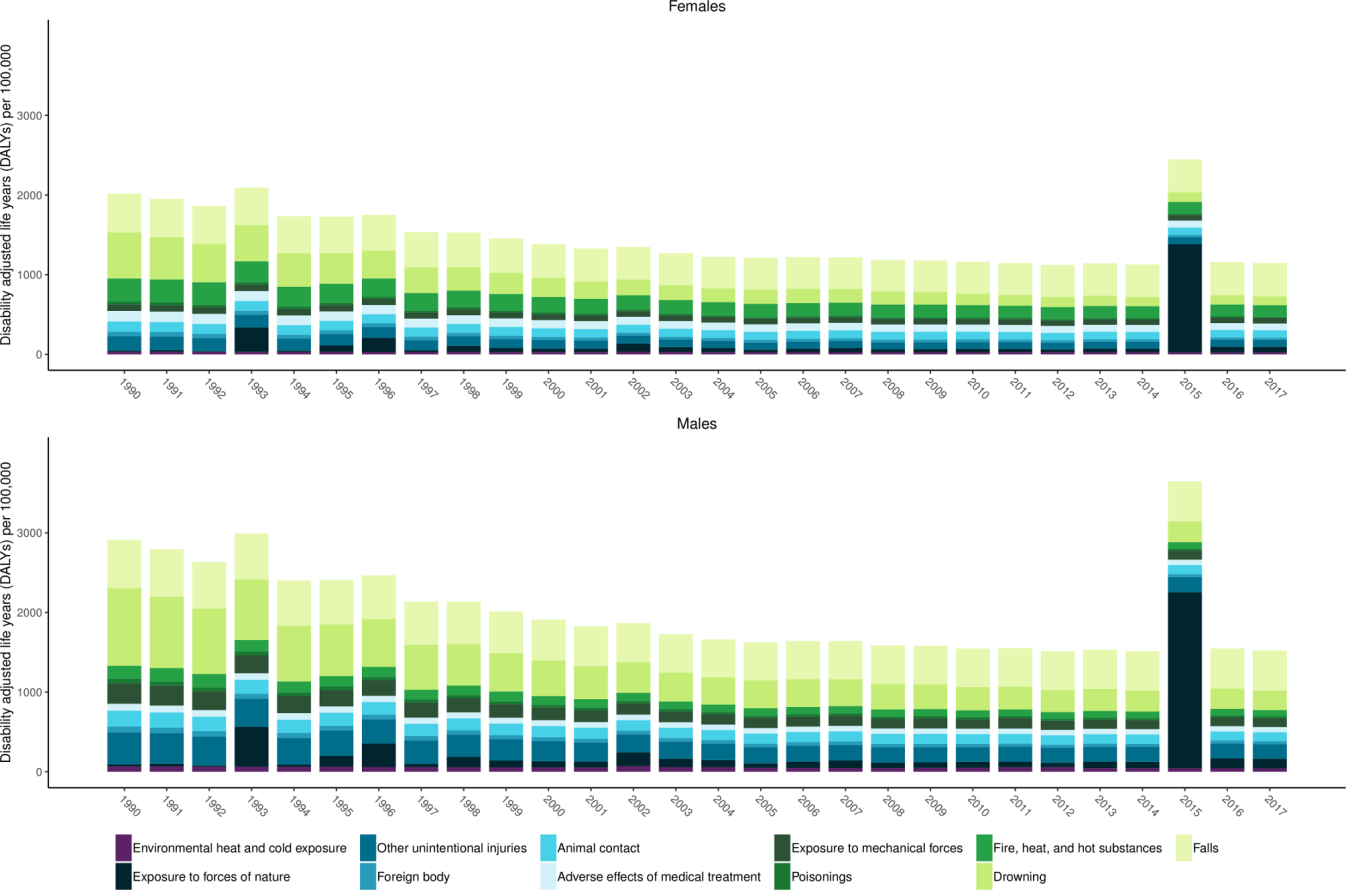
Age-standardised DALYs per 100 000 by year for females and males for level 3 unintentional injury subcauses in the Global Burden of Disease Study (GBD) cause hierarchy between 1990 and 2017.

Figure 4 shows age-standardised DALYs per 100 000 for unintentional injuries by subtype between 1990 and 2017 for females and males. This figure shows several key trends. First, falls and drowning are predominant causes of unintentional injury DALYs across the time period. Second, the health loss toll of exposure to forces of nature can be substantial. Third, the gap between males and females in terms of DALYs is smaller for unintentional injuries than for transport injuries. The large increases in DALYs in 2015 correspond to the Nepal earthquake, while in 1993 there was a large increase due to flooding and landslides in South Central Nepal.

Associated results are provided in online supplementary appendix table 1.

These results show how in 1990, there were 7402 (5616 to 9162) deaths due to unintentional injuries in Nepal causing 451 366 (327 272 to 578 048) YLLs and 59 629 (44 121 to 76 776) YLDs forming a total of 510 995 (385 060 to 639 142) DALYs. By 2017, there were 6465 (5250 to 7642) deaths due to unintentional injuries in Nepal causing 240 316 (188 944 to 294 055) YLLs and 117 911 (88 529 to 151 651) YLDs forming a total of 358 227 (295 708 to 424 802) DALYs.

Falls were the cause of 11.84% (9.13 to 15.79%) of all injury mortality in 1990 and rose to 17.14% (13.25 to 23.60%) in 2017.

### Intentional injuries

**Figure 5:**
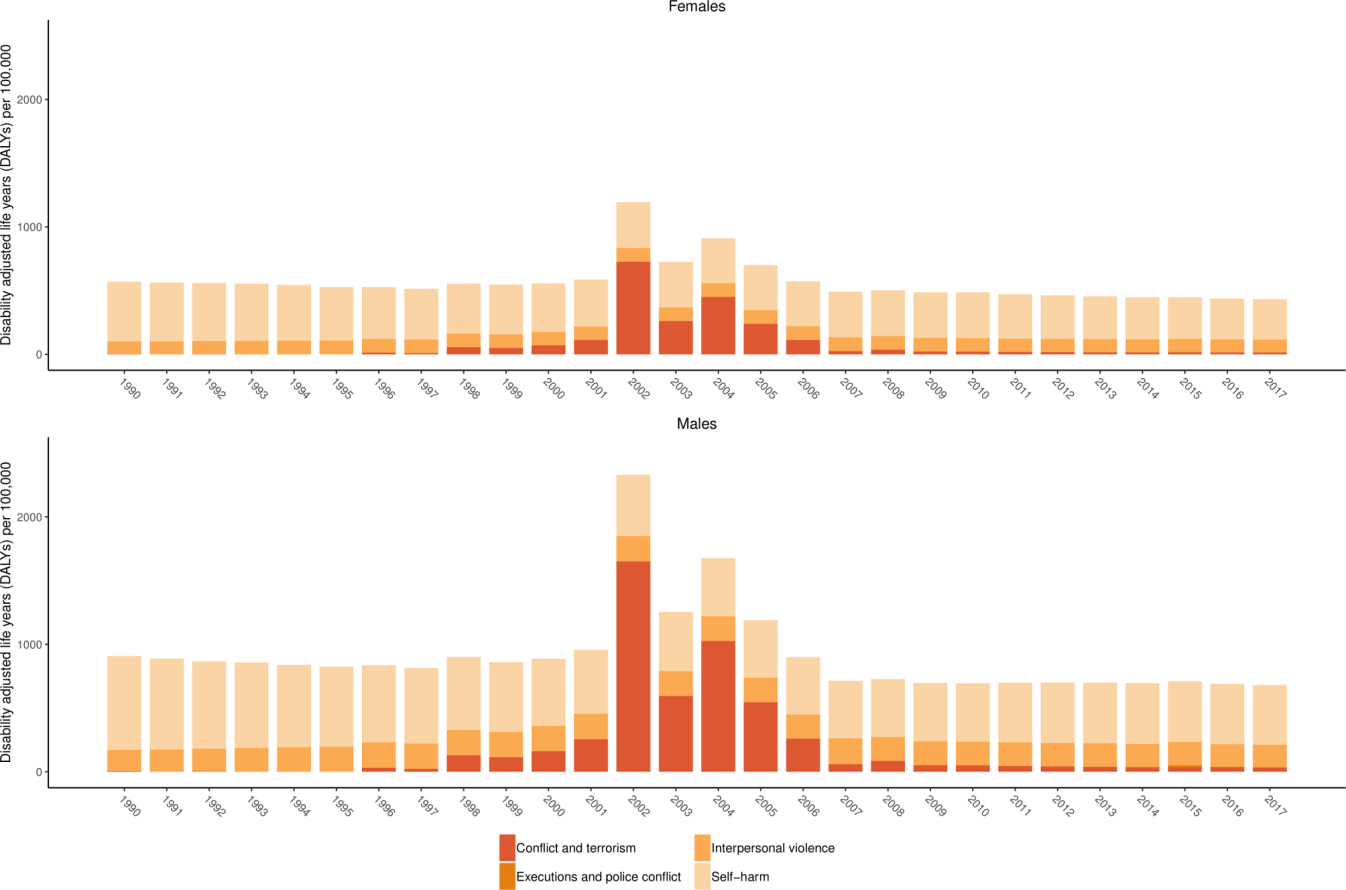
Age-standardised DALYs per 100 000 by year for females and males for level 3 self-harm and interpersonal violence subcauses in the Global Burden of Disease Study (GBD) cause hierarchy between 1990 and 2017.

Figure 5 shows age-standardised DALYs for self-harm and interpersonal violence by subtype between 1990 and 2017 for females and males. This figure shows the large increases in DALYs due to conflict and terrorism attributed to the Maoist conflict during the period from 1996 to 2006.

The figure also shows how, similar to unintentional injuries, the gap between age-standardised DALYs per 100 000 is smaller between females and males than in the case of transport injuries. Associated results are provided in online supplementary appendix table 1, which shows how in 1990 there were 2200 (1521 to 2870) deaths due to self-harm and interpersonal violence in Nepal causing 122 594 (82 184 to 159 287) YLLs and 8886 (6782 to 11 330) YLDs forming a total of 131 480 (91 538 to 168 953) DALYs. By 2017, there were 2842 (2081 to 3519) deaths due to self-harm and interpersonal violence in Nepal causing 144 731 (103 973 to 181 855) YLLs and 22 707 (17 518 to 28 735) YLDs forming a total of 167 438 (125 687 to 203 650) DALYs.

Self-harm was the cause of 14.07% (10.80% to 18.64%) of all-injury mortality in 1990 and slightly decreased to 13.94% (10.62% to 18.90%) in 2017.

## Discussion

Since 1990, injury mortality rates and overall age-standardised rates of DALYs due to injuries have declined, but their proportion in terms of overall deaths and DALYs has increased over time. The magnitude and pattern of injury burden varies over time by injury type, sex and age group. This study revealed that young and middle-aged adults experience a greater burden of injuries than other age groups, which is an important observation given that injury burden may adversely affect the social and economic development of a country. The expected increase in life expectancy of the population in Nepal and the increasing number of people with disability suggest that the injury burden will continue to rise in the coming decades.^31^

There are several factors that are likely to have affected the burden of injuries in Nepal. First, former research has demonstrated deficits in the availability of trauma care across several domains of emergency medical care.^14^ Injuries have been recently acknowledged as a public health problem by the National Health Policy 2014,^32^ and the NHSS Implementation Plan 2016–2021^17^ has set targets by 2020 to reduce road injuries. Due to the historic absence of injury-focused policy and strategies, however, injury prevention initiatives may take time to gain traction.

Second, limited availability of injury burden data may have affected the visibility of injury as a public health issue in Nepal. While the GBD study maximises the use of available data, there are generally limited data available for injury burden measurement, and data sources that do exist are often incomplete. For example, the Annual Report of the Department of Health Services (DoHS) is the main source of data for estimating the burden of disease and injuries within Nepal, but information and statistics used in DoHS reports are based on the data collected through the health management information system from health facilities that may have incomplete records of inpatient and outpatient care. Injury recording and reporting are usually performed by health facilities using the International Classification of Diseases Tenth Revision (ICD-10) classification with inconsistency in utilisation of ICD codes. In fiscal year 2016–2017, ICD-10 code V89 (motor or non-motor vehicle accident, type of vehicle unspecified) was the ninth leading cause of death in inpatients, while ICD-10 code T14 (injury of unspecified body region) was the fourth leading cause of morbidities among inpatients, and ‘falls/injuries/fractures’ was the fifth leading cause for outpatient consultation. These three categories of classification are ambiguous and do not give specific information about types of injuries.^33^ Classification ambiguity could also affect the visibility of injury burden. Moreover, Nepal has no formal injury surveillance system, which may be an important investment in measuring and responding to future injury burden.^7^ In fact, only two national-level studies on injuries were conducted in Nepal between 1995 and 2017.^34 35^ In addition, research on the socioeconomic impact of injuries, the economic evaluation of injury interventions and on vulnerable road users has been limited, which may also contribute to limited focus on injury as an important public health topic in Nepal. Future injury burden estimation in Nepal may also benefit from the addition of crime and police data.

Third, the public understanding of the term ‘injury’ with associated causes and consequences in Nepal differs from the well-accepted meaning of injury in health research. Traditionally, injuries in Nepal are often considered to be ‘accidents’ and as a result are cited as an inevitable act because of bad coincidence or ill fate. Injury events are called *Chotpatak* in both formal and informal Nepali language, meaning discernible ‘trauma’, ‘wound’, ‘hurt’, ‘bruise’, ‘bite’ or ‘blow’, except for intentional assaults.^36^ We speculate that realisation by families and the community that the risk of injury should be viewed similarly to the risk of other diseases is important for injury prevention since it could drive behaviour in terms of prevention and care seeking after an injury. It seems plausible that such perceptions could also cause injuries to be under-reported in local and national-level data recording systems.

### Transport injuries

Transport injuries are an important part of injury burden both globally and in Nepal. In this study, we showed that in 2017 transport injury was a leading cause of death for many age groups, particularly adult age groups. 37 GBD 2017 estimates for age-standardised 'transport injuries' mortality rates in Nepal (29.86 per 100 000 population) were higher than the WHO estimates for Nepal of 15.9 per 100 000 in 2016.^37^ However, the WHO estimates of roadinjuries only include pedestrian, cyclist, motorcyclists, motor vehicles and other road injuries and the informationfor "other transport injuries" might not havebeen accounted for. Transport injuries killed more people in the 15–49 years age group than the burden of HIV/AIDS, tuberculosis (TB) and malaria for the same age in the same period in Nepal.^2–7 38^ GBD estimates also show that 'road injuries' killed 3394 people in 2017 whereas HIV, TB and malaria together kill 3120 people aged 15–49 years. WHO estimates also show that transport injuries are a leading cause of death among those aged 15–29 years.^37^ The proportion of deaths caused by injuries on the roads among the adult population is consistent with the recent systematic review and secondary data analysis of traffic police records in Nepal.^8^ An epidemiological study using secondary data from emergency departments in 11 tertiary hospitals of Nepal conducted by the Nepal Health Research Council also reported a similar trend of transport injuries.^7^

There are several possible reasons for the increased incidence of and mortality due to transport injuries in Nepal. Nepal has challenging road conditions in urban settings with a lack of crosswalks and few traffic lights as well as in rural settings with difficult topography and engineering challenges. The growing number of motorised vehicles along with higher vehicle speeds, poor quality roads that lack pedestrian walkways, non-compliance of traffic rules and lack of road safety awareness among road users have been suggested as possible explanations for increasing road traffic deaths.^7^ As noted by recent studies, distracted driving such as using mobile phones while driving^39^ and driving under the influence of drugs and alcohol^40^ may play an important role in the risk of transport injury, and one recent study on the effect of breathalyser checks found a decreased rate of road traffic crashes and case fatalities in Katmandu.^41^ According to the Department of Transport Management, there were a total of 2 783 428 registered motorised vehicles (cumulative until 2016–2017)^42^ and 2 297 141 registered licence holders (cumulative until 2016–2017 as calculated by the authors of this study). In addition, over 80 000 new driving licences were issued in the years 2014–2015. It may be that road network capacity, policy changes and enforcement of road safety laws—for example, urban speed limits and laws requiring the use of seat belts and motorcycle helmets, which were implemented in the 1990s—are not keeping pace with growth in vehicle density. In light of these developments, further examination into the role of road injury prevention interventions in averting injury incidence as well as the role of healthcare and rehabilitation services in reducing road injury-related death and disability is warranted.

### Unintentional injuries

In GBD, this injury category includes falls, drowning, burns, poisonings, exposure to mechanical forces, adverse effects of medical treatment, animal related, foreign body, environmental heat and cold exposure, and forces of nature (for more details, please refer to table 2). A large baseline survey conducted in rural Bangladesh found that the rates for both fatal and non-fatal fall injuries were highest among older age groups (above 64 years).^43^ Given scant data on fall injuries in LMICs, the factors of fall injuries in the elderly in these regions, including Nepal, are largely unknown, but the WHO has stated that occupational, alcohol and substance use, socioeconomic factors, medications, underlying medical conditions such as loss of balance and poor vision, and environmental factors are all associated with risk of fall injuries.^44^ Other research has suggested that population growth and an ageing population contribute to the increase in fall injuries.^5^

Between 1990 and 2017, the number of unintentional injury-related deaths decreased, which was mainly due to the reductions in the number of deaths from drowning and fires, heat and hot substances.^5^ However, we did not identify the interventions during the period of this study that were aimed at decreasing drowning and fire-related deaths in Nepal. Further investigations are required to understand the decline in trends of drowning and fire-related deaths in Nepal. For falls and fires, GBD 2017 found that the mortality rate was higher in females than in males.^5^ This trend contrasts with a study conducted by Fayyaz *et al* 45 in Pakistan as well as WHO estimates.^46^ This finding may be partly due to Nepalese females being more exposed to injury risk from household chores, and in particular to cooking and to collecting firewood and animal fodder, which can involve climbing trees. Instituting a national burn registry would support our understanding of the trends in fire and burn-related injuries in Nepal.

There was a sudden increase in deaths in 2015 that can be attributed to the massive 2015 earthquake and its aftershocks. As natural disasters of this type are largely unavoidable, continuing to increase more resources to infrastructure improvements and emergency medical systems could help prevent future morbidity and mortality from such disasters and would be aligned with trauma care system guidance provided by WHO.^46^

### Intentional injuries

In the GBD cause hierarchy, intentional injuries include self-harm, interpersonal violence, conflict and terrorism (for more details, please refer to table 2). Self-harm and interpersonal violence have consistently formed an important component of injury burden with particularly notable contribution to injury DALYs in males in the young to middle adult age ranges. While the GBD study design incorporated modelling techniques to adjust for under-reporting and misclassification of the cause of death, it is possible that this burden is still underestimated if these injuries are systematically under-reported, particularly among females of reproductive age, which previous research has also cited as a critical issue.^47 48^ Improving the quality and completeness of intentional injury data may require changes to enable front-line staff to better record known self-harm, assault and gender-based violence injuries. For example, reducing the stigma associated with reporting gender-based violence, improved training on how to record intentional harm and how to use a full range of classification options for intentional harm. Measuring the burden of sexual violence would benefit from identifying and adding additional data sources to future updates to the GBD study. Notably, we found striking trends in death rates due to conflict. Some deaths can be related to an unstable political environment. For example, large increases in death and disability in the early 2000s correspond to the Maoists’ armed conflict in Nepal between 1996 and 2006.^49^

### Limitations

This study has several limitations. First, GBD 2017 results were the only source of estimates used in this paper due to the aforementioned issue of limited national sources on fatal and non-fatal injury burden in Nepal. This means estimates presented here are subject to the limitations of GBD 2017, which are discussed in more detail elsewhere.^1–6^ Second, estimation of the burden of injury was limited to standard epidemiological measures, meaning economic and social burdens were not considered. These exclusions may mean this study underestimated the true injury burden in Nepal. Third, GBD 2017 lacked data on cause of death in Nepal, so cause-specific mortality estimates were driven by covariates—such as sociodemographic index, vehicles per capita and alcohol use—and by the modelling framework, which borrows strength from neighbouring locations that may have different injury profiles. In the future, it would strengthen GBD estimates for Nepal to add data on causes of death.

## Conclusion

Injuries remain an important cause of morbidity and mortality in Nepal, with transport injuries and falls being prominent causes of death in the country. Intentional injuries also contribute a considerable burden. There is a need for federal, provincial and local governments to prioritise injury prevention as a public health agenda and to cultivate cross-cutting, intersectorial efforts in order to develop and implement injury prevention interventions. At the population level, raising public awareness of injuries needs to be a part of any preventative initiative to address the burden of injury. More research funding is duly needed to identify the burden of injuries, their causes and their consequences to establish evidence-based, country-specific interventions.

**What is already known on the subject**

- Injuries are an important cause of morbidity and mortality in low/middle-income countries.
- People in Nepal are at risk of preventable injury at home, in the workplace and on the roads in addition to natural disasters (earthquakes, landslides and monsoon flooding).
- No reliable and comprehensive national estimates for trends in injury burden in Nepal have been published previously.

**What this study adds**

- This study is the first to report estimates of injury burden in Nepal using Global Burden of Disease Study (GBD) data and estimated that in 2017, there were 16 831 (13 323 to 20 579) deaths caused by injuries, compared with 13 567 (10 466 to 16 061) deaths caused by injuries in 1990.
- In 2017, transport injuries killed more people aged 15–49 years than from HIV, tuberculosis and malaria combined together; adult age groups sustained the greatest proportion of transport injury disability-adjusted life years (DALYs).
- Although, since 1990, there has been a notable decrease in the rates of deaths and DALYs of injury, its proportion to overall mortality has continuously increased over time and varies widely by cause-specific injuries, sex and age groups.
- GBD resources can be used to develop country-level disease-specific summaries of potential interest to researchers and policymakers.
