# Supplementary material for: Burden of injuries in Nepal, 1990–2017: findings from the Global Burden of Disease Study 2017
Source: Inj Prev. 2020 Jan 8;26(Suppl 1):i57–66. doi: 10.1136/injuryprev-2019-043309 (PMC7571348; doi:10.1136/injuryprev-2019-043309)
Supplement: Supplementary data [file injuryprev-2019-043309supp003.pdf]

Table 1: Death, YLL, incidence, prevalence, YLD, and DALY counts and rates per 100,000 for all injuries in Nepal in 1990 and 2017

Cause	Deaths: All-age counts (95% UI) 1990	Deaths: All-age counts (95% UI) 2017	Deaths: Age-standardised rates per 100,000 (95% UI) 1990	Deaths: Age-standardised rates per 100,000 (95% UI) 2017	Years of life lost: All-age counts (95% UI) 1990	Years of life lost: All-age counts (95% UI) 2017	Years of life lost: Age-standardised rates per 100,000 (95% UI) 1990	Years of life lost: Age-standardised rates per 100,000 (95% UI) 2017
**All injuries**	**13 567 (10 466 to 16 061)**	**16 831 (13 323 to 20 579)**	**88.9 (71.5 to 105.3)**	**70.3 (56.7 to 85.1)**	**791 622 (581 823 to 958 876)**	**715 602 (540 423 to 894 816)**	**3 911.8 (2 974.8 to 4 636.4)**	**2 498.6 (1 899.6 to 3 120.4)**
**Transport injuries**	**3 965 (2 724 to 5 282)**	**7 524 (4 480 to 11 214)**	**25.5 (18.0 to 34.4)**	**29.4 (17.3 to 43.6)**	**217 661 (144 877 to 288 126)**	**330 555 (200 266 to 507 611)**	**1 178.6 (800.5 to 1 576.7)**	**1 144.1 (682.9 to 1 739.1)**
Road injuries	3 667 (2 490 to 4 897)	6 787 (3 920 to 10 235)	23.6 (16.6 to 32.0)	26.6 (15.2 to 39.3)	201 340 (132 752 to 266 882)	296 686 (173 127 to 454 931)	1 089.4 (734.9 to 1 461.0)	1 029.7 (599.8 to 1 569.3)
Pedestrian road injuries	1 946 (1 238 to 2 863)	3 588 (1 649 to 6 625)	12.6 (8.2 to 18.8)	14.7 (6.8 to 26.5)	108 580 (67 245 to 159 976)	145 251 (64 647 to 278 305)	565.7 (354.9 to 841.5)	519.6 (233.9 to 981.1)
Cyclist road injuries	130 (65 to 240)	473 (140 to 1 409)	0.9 (0.5 to 1.7)	1.9 (0.5 to 5.7)	6 537 (3 139 to 11 839)	19 948 (5 726 to 58 441)	38.6 (19.3 to 70.9)	69.5 (20.1 to 207.3)
Motorcyclist road injuries	738 (428 to 1 199)	1 309 (624 to 2 616)	4.6 (2.7 to 7.6)	4.6 (2.1 to 9.4)	40 358 (23 014 to 64 872)	65 885 (32 366 to 130 622)	228.7 (132.7 to 373.6)	216.8 (103.1 to 432.4)
Motor vehicle road injuries	815 (405 to 1 463)	1 346 (534 to 2 873)	5.3 (2.7 to 9.8)	5.1 (2.0 to 10.9)	43 995 (21 226 to 76 984)	62 822 (26 043 to 134 169)	246.0 (122.1 to 440.4)	213.8 (89.0 to 460.3)
Other road injuries	37 (12 to 74)	70 (23 to 202)	0.3 (0.1 to 0.6)	0.3 (0.1 to 0.9)	1 870 (583 to 3 612)	2 779 (967 to 8 058)	10.4 (3.5 to 21.1)	10.1 (3.4 to 29.6)
Other transport injuries	299 (177 to 439)	737 (246 to 1 361)	1.9 (1.1 to 2.8)	2.8 (0.9 to 5.1)	16 321 (9 622 to 24 326)	33 869 (11 225 to 62 921)	89.2 (52.7 to 132.0)	114.3 (38.0 to 211.7)
**Unintentional injuries**	**7 402 (5 616 to 9 162)**	**6 465 (5 250 to 7 642)**	**49.9 (39.7 to 60.8)**	**30.8 (25.0 to 36.1)**	**451 366 (327 272 to 578 048)**	**240 316 (188 944 to 294 055)**	**2 051.6 (1 540.7 to 2 540.7)**	**885.9 (703.1 to 1 076.1)**
Falls	1 603 (1 135 to 2 104)	2 911 (2 253 to 3 619)	18.8 (13.4 to 26.4)	16.4 (12.8 to 20.1)	60 994 (41 190 to 83 454)	72 434 (52 704 to 95 796)	409.1 (286.5 to 534.9)	310.9 (231.8 to 399.9)
Drowning	2 748 (1 874 to 3 695)	941 (715 to 1 143)	11.3 (8.2 to 14.6)	3.4 (2.7 to 4.1)	212 147 (140 575 to 290 466)	53 523 (39 116 to 67 787)	773.2 (528.7 to 1 037.6)	174.2 (127.3 to 218.9)
Fire, heat, and hot substances	522 (350 to 724)	373 (256 to 538)	3.3 (2.3 to 4.6)	1.5 (1.1 to 2.1)	31 186 (19 268 to 43 802)	17 258 (11 196 to 26 182)	150.1 (99.9 to 208.7)	59.4 (38.7 to 89.0)
Poisonings	152 (66 to 244)	85 (58 to 129)	0.7 (0.4 to 1.1)	0.3 (0.2 to 0.5)	10 903 (4 295 to 18 456)	4 929 (3 159 to 7 878)	43.1 (18.9 to 68.9)	16.0 (10.3 to 25.7)
Poisoning by carbon monoxide	22 (11 to 30)	20 (14 to 30)	0.2 (0.1 to 0.2)	0.1 (0.1 to 0.1)	1 235 (622 to 1 740)	973 (609 to 1 509)	6.3 (3.2 to 8.7)	3.2 (2.1 to 4.9)
Poisoning by other means	130 (54 to 217)	64 (43 to 100)	0.6 (0.3 to 0.9)	0.2 (0.2 to 0.3)	9 668 (3 640 to 16 893)	3 955 (2 475 to 6 334)	36.8 (15.5 to 61.2)	12.8 (8.0 to 20.4)
Exposure to mechanical forces	445 (324 to 586)	317 (243 to 401)	2.9 (2.2 to 3.8)	1.3 (1.0 to 1.6)	25 530 (18 001 to 34 287)	14 206 (10 605 to 18 284)	128.0 (92.6 to 169.7)	49.2 (36.8 to 63.1)
Unintentional firearm injuries	41 (22 to 67)	24 (16 to 32)	0.3 (0.2 to 0.5)	0.1 (0.1 to 0.1)	2 118 (1 047 to 3 460)	935 (615 to 1 284)	11.8 (6.2 to 19.3)	3.4 (2.2 to 4.6)
Other exposure to mechanical forces	404 (294 to 539)	293 (225 to 372)	2.6 (1.9 to 3.4)	1.2 (0.9 to 1.5)	23 412 (16 469 to 31 816)	13 271 (9 900 to 17 178)	116.3 (84.1 to 155.5)	45.8 (34.5 to 59.0)
Adverse effects of medical treatment	411 (319 to 506)	605 (473 to 744)	3.8 (3.0 to 4.9)	2.9 (2.4 to 3.6)	17 908 (12 454 to 24 127)	18 761 (14 304 to 23 709)	108.8 (84.0 to 134.3)	74.5 (57.6 to 92.8)
Animal contact	462 (224 to 639)	440 (192 to 661)	2.8 (1.4 to 3.8)	1.7 (0.8 to 2.5)	27 717 (13 018 to 39 312)	21 149 (8 925 to 32 965)	133.6 (64.1 to 185.9)	72.2 (30.8 to 112.2)
Venomous animal contact	429 (210 to 592)	407 (173 to 622)	2.6 (1.3 to 3.5)	1.6 (0.7 to 2.4)	25 588 (12 044 to 35 656)	19 564 (8 130 to 30 766)	124.4 (60.4 to 172.2)	66.7 (27.8 to 105.0)
Non-venomous animal contact	33 (13 to 95)	33 (14 to 51)	0.2 (0.1 to 0.5)	0.1 (0.1 to 0.2)	2 128 (766 to 6 745)	1 585 (698 to 2 459)	9.2 (3.7 to 26.8)	5.5 (2.4 to 8.5)
Foreign body	208 (152 to 285)	127 (100 to 174)	1.0 (0.8 to 1.4)	0.6 (0.5 to 0.8)	15 428 (10 545 to 21 887)	6 075 (4 470 to 8 839)	54.5 (39.6 to 75.0)	22.1 (16.5 to 31.7)
Pulmonary aspiration and foreign body in airway	179 (128 to 255)	112 (89 to 157)	0.9 (0.7 to 1.2)	0.5 (0.4 to 0.7)	13 432 (8 952 to 19 791)	5 394 (3 912 to 8 023)	46.6 (33.1 to 66.6)	19.7 (14.6 to 28.7)
Foreign body in eyes	-	-	-	-	-	-	-	-
Foreign body in other body part	28 (15 to 46)	14 (10 to 20)	0.1 (0.1 to 0.2)	0.1 (0.0 to 0.1)	1 995 (837 to 3 467)	681 (444 to 990)	7.9 (4.2 to 12.7)	2.4 (1.6 to 3.4)
Environmental heat and cold exposure	102 (37 to 144)	91 (34 to 129)	0.9 (0.3 to 1.4)	0.4 (0.2 to 0.6)	4 565 (1 552 to 7 475)	2 770 (1 035 to 4 084)	26.8 (9.8 to 38.3)	11.2 (4.2 to 16.3)
Exposure to forces of nature	30 (29 to 31)	154 (149 to 159)	0.2 (0.2 to 0.2)	0.5 (0.5 to 0.6)	1 823 (1 748 to 1 905)	8 532 (8 200 to 8 891)	8.8 (8.5 to 9.1)	27.6 (26.6 to 28.6)
Other unintentional injuries	719 (472 to 910)	423 (285 to 538)	4.2 (2.8 to 5.2)	1.6 (1.1 to 2.0)	43 166 (27 363 to 55 020)	20 680 (13 707 to 26 826)	215.6 (138.7 to 273.7)	68.7 (45.7 to 88.6)
**Self-harm and interpersonal violence**	**2 200 (1 521 to 2 870)**	**2 842 (2 081 to 3 519)**	**13.5 (9.7 to 17.6)**	**10.1 (7.4 to 12.4)**	**122 594 (82 184 to 159 287)**	**144 731 (103 973 to 181 855)**	**681.7 (467.3 to 893.0)**	**468.6 (334.3 to 587.2)**
Self-harm	1 909 (1 290 to 2 551)	2 341 (1 737 to 2 835)	11.8 (8.3 to 15.7)	8.4 (6.3 to 10.1)	105 264 (68 824 to 140 136)	117 209 (85 342 to 144 638)	591.9 (398.2 to 791.3)	380.9 (276.1 to 466.7)
Self-harm by firearm	172 (79 to 301)	148 (84 to 235)	1.0 (0.5 to 1.8)	0.5 (0.3 to 0.8)	9 791 (4 323 to 17 374)	7 882 (4 360 to 13 015)	54.1 (24.4 to 95.0)	24.9 (13.8 to 40.4)
Self-harm by other specified means	1 736 (1 198 to 2 330)	2 192 (1 637 to 2 660)	10.8 (7.7 to 14.5)	7.9 (6.0 to 9.5)	95 473 (63 487 to 128 363)	109 327 (80 218 to 133 669)	537.8 (369.6 to 724.0)	355.9 (259.1 to 435.1)
Interpersonal violence	276 (190 to 362)	487 (193 to 673)	1.6 (1.1 to 2.1)	1.6 (0.7 to 2.3)	16 350 (11 123 to 21 392)	26 698 (9 736 to 37 097)	85.1 (57.8 to 111.4)	85.1 (32.5 to 118.2)
Assault by firearm	34 (9 to 54)	79 (10 to 135)	0.2 (0.1 to 0.3)	0.3 (0.0 to 0.4)	1 906 (487 to 3 083)	4 289 (522 to 7 480)	10.6 (2.7 to 17.0)	13.7 (1.7 to 23.5)
Assault by sharp object	45 (22 to 73)	95 (25 to 150)	0.3 (0.1 to 0.4)	0.3 (0.1 to 0.5)	2 472 (1 115 to 4 072)	5 000 (1 161 to 8 103)	14.0 (6.7 to 23.1)	16.3 (3.9 to 26.0)
Sexual violence	-	-	-	-	-	-	-	-
Assault by other means	198 (138 to 292)	312 (154 to 452)	1.1 (0.8 to 1.7)	1.0 (0.5 to 1.5)	11 972 (8 265 to 17 418)	17 409 (7 915 to 25 371)	60.6 (42.0 to 89.6)	55.2 (26.0 to 80.1)
Conflict and terrorism	14 (14 to 14)	0 (0 to 0)	0.1 (0.1 to 0.1)	0.0 (0.0 to 0.0)	889 (871 to 906)	0 (0 to 0)	4.2 (4.1 to 4.3)	0.0 (0.0 to 0.0)
Executions and police conflict	2 (1 to 2)	15 (14 to 15)	0.0 (0.0 to 0.0)	0.0 (0.0 to 0.1)	91 (66 to 108)	824 (788 to 855)	0.4 (0.3 to 0.5)	2.6 (2.5 to 2.7)

Cause	Incidence: All-age counts (95% UI) 1990	Incidence: All-age counts (95% UI) 2017	Incidence: Age-standardised rates per 100,000 (95% UI) 1990	Incidence: Age-standardised rates per 100,000 (95% UI) 2017	Prevalence: All-age counts (95% UI) 1990	Prevalence: All-age counts (95% UI) 2017	Prevalence: Age-standardised rates per 100,000 (95% UI) 1990	Prevalence: Age-standardised rates per 100,000 (95% UI) 2017
**All injuries**	**832 671 (794 609 to 877 773)**	**1 493 655 (1 407 015 to 1 589 728)**	**4 725.2 (4 525.8 to 4 946.0)**	**5 151.7 (4 877.6 to 5 447.1)**	**1 929 256 (1 833 918 to 2 026 453)**	**4 282 938 (4 046 131 to 4 546 052)**	**13 272.7 (12 687.9 to 13 913.8)**	**15 793.3 (14 997.4 to 16 713.0)**
**Transport injuries**	**92 117 (82 284 to 103 083)**	**186 719 (165 111 to 214 229)**	**488.6 (441.9 to 539.5)**	**621.2 (552.2 to 703.7)**	**263 759 (237 329 to 289 884)**	**590 541 (533 911 to 645 954)**	**1 883.1 (1 713.0 to 2 044.3)**	**2 269.3 (2 065.3 to 2 472.5)**
Road injuries	57 705 (49 864 to 65 770)	132 834 (113 152 to 156 543)	321.5 (279.8 to 363.8)	437.2 (374.8 to 507.9)	139 559 (129 457 to 150 900)	353 306 (325 678 to 383 730)	1 047.5 (970.4 to 1 137.7)	1 382.3 (1 275.8 to 1 504.2)
Pedestrian road injuries	19 281 (15 930 to 22 893)	38 122 (30 699 to 47 029)	106.3 (88.6 to 124.2)	125.8 (103.1 to 153.5)	54 514 (47 753 to 63 984)	120 188 (103 743 to 142 847)	405.2 (355.5 to 474.2)	469.7 (406.9 to 559.4)
Cyclist road injuries	11 910 (9 599 to 14 921)	29 643 (23 241 to 37 754)	64.5 (51.8 to 79.2)	95.9 (75.4 to 120.8)	20 593 (17 346 to 24 042)	57 229 (48 077 to 67 367)	151.6 (127.4 to 176.4)	221.2 (185.9 to 259.2)
Motorcyclist road injuries	16 530 (13 715 to 19 948)	40 603 (32 549 to 50 417)	94.6 (79.0 to 113.5)	133.1 (107.8 to 163.4)	49 391 (42 850 to 56 753)	133 810 (114 982 to 155 224)	376.5 (325.2 to 433.9)	524.7 (451.3 to 607.7)
Motor vehicle road injuries	5 762 (4 547 to 7 218)	13 910 (10 475 to 18 024)	31.9 (25.1 to 39.8)	46.1 (35.0 to 58.9)	8 855 (7 762 to 10 170)	24 244 (21 233 to 28 241)	66.4 (58.4 to 76.5)	95.4 (83.2 to 111.5)
Other road injuries	4 222 (3 146 to 5 469)	10 555 (7 699 to 13 787)	24.3 (18.2 to 30.9)	36.2 (26.9 to 46.7)	6 207 (5 189 to 7 489)	17 835 (14 945 to 21 499)	47.8 (40.1 to 57.6)	71.3 (59.7 to 85.7)
Other transport injuries	34 412 (28 330 to 42 092)	53 886 (44 407 to 65 821)	167.2 (143.1 to 196.4)	184.0 (153.6 to 221.2)	124 200 (102 807 to 146 840)	237 235 (197 144 to 280 038)	835.6 (705.5 to 970.6)	887.0 (748.7 to 1 037.7)
**Unintentional injuries**	**704 441 (667 076 to 745 627)**	**1 248 734 (1 166 353 to 1 337 130)**	**4 046.1 (3 847.4 to 4 268.3)**	**4 338.4 (4 083.8 to 4 622.5)**	**1 104 170 (1 049 920 to 1 180 482)**	**2 437 466 (2 278 371 to 2 642 009)**	**8 092.6 (7 691.0 to 8 655.0)**	**9 301.9 (8 748.8 to 10 028.7)**
Falls	175 311 (154 909 to 199 139)	349 043 (302 584 to 403 990)	1 114.9 (1 001.3 to 1 246.6)	1 290.9 (1 135.8 to 1 467.5)	305 162 (272 422 to 342 160)	677 787 (600 780 to 764 528)	2 386.8 (2 149.4 to 2 666.5)	2 725.9 (2 434.8 to 3 064.4)
Drowning	1 281 (1 140 to 1 433)	1 210 (1 050 to 1 415)	6.3 (5.8 to 6.9)	4.3 (3.8 to 4.9)	5 463 (4 607 to 6 389)	6 181 (5 359 to 7 095)	37.3 (32.1 to 42.8)	23.9 (21.0 to 27.4)
Fire, heat, and hot substances	19 398 (16 373 to 23 241)	30 694 (25 026 to 37 840)	99.8 (85.6 to 117.7)	97.3 (80.2 to 118.6)	153 731 (134 200 to 175 569)	274 906 (236 375 to 318 921)	1 081.9 (947.1 to 1 237.6)	1 029.3 (887.9 to 1 191.8)
Poisonings	4 248 (3 171 to 5 634)	7 571 (5 495 to 10 075)	17.5 (13.5 to 22.6)	23.4 (17.2 to 30.8)	3 326 (2 791 to 3 998)	7 433 (6 271 to 8 929)	19.9 (16.9 to 23.8)	25.8 (21.9 to 30.8)
Poisoning by carbon monoxide	1 109 (753 to 1 641)	2 019 (1 293 to 3 036)	4.7 (3.3 to 6.7)	6.3 (4.1 to 9.3)	724 (585 to 891)	1 616 (1 301 to 1 997)	4.4 (3.6 to 5.4)	5.7 (4.6 to 7.0)
Poisoning by other means	3 139 (2 263 to 4 271)	5 552 (3 945 to 7 682)	12.8 (9.5 to 16.9)	17.1 (12.2 to 23.4)	2 603 (2 064 to 3 203)	5 817 (4 649 to 7 256)	15.5 (12.4 to 19.0)	20.1 (16.2 to 25.0)
Exposure to mechanical forces	91 777 (79 308 to 105 102)	143 989 (120 714 to 172 104)	485.6 (427.5 to 553.5)	484.1 (409.9 to 576.3)	161 316 (142 652 to 182 935)	300 621 (263 046 to 346 003)	1 153.9 (1 024.8 to 1 305.3)	1 142.6 (1 000.3 to 1 311.4)
Unintentional firearm injuries	2 635 (1 860 to 3 611)	3 688 (2 514 to 5 155)	12.2 (8.9 to 16.2)	12.1 (8.4 to 16.9)	7 421 (6 174 to 9 066)	12 698 (10 638 to 15 186)	49.4 (41.5 to 58.7)	47.1 (39.5 to 55.7)
Other exposure to mechanical forces	89 143 (77 102 to 102 143)	140 301 (117 777 to 167 559)	473.3 (417.6 to 540.0)	472.0 (400.6 to 562.0)	153 894 (134 828 to 175 603)	287 922 (248 748 to 333 494)	1 104.5 (977.0 to 1 253.5)	1 095.5 (951.5 to 1 264.8)
Adverse effects of medical treatment	16 136 (13 826 to 18 542)	31 121 (25 879 to 36 660)	96.4 (84.3 to 109.2)	111.0 (93.5 to 128.7)	1 235 (963 to 1 531)	2 382 (1 814 to 2 964)	7.4 (5.8 to 9.1)	8.5 (6.5 to 10.5)
Animal contact	198 095 (171 357 to 227 931)	345 100 (292 280 to 408 311)	1 101.0 (966.0 to 1 252.7)	1 157.9 (994.2 to 1 351.3)	120 824 (105 890 to 136 955)	238 886 (206 541 to 275 095)	880.2 (772.8 to 992.0)	912.7 (792.0 to 1 041.1)
Venomous animal contact	107 917 (88 915 to 129 731)	206 319 (167 193 to 253 862)	600.1 (497.9 to 713.0)	684.7 (558.3 to 828.7)	71 136 (58 920 to 84 097)	160 099 (132 153 to 190 750)	519.4 (431.1 to 608.2)	610.0 (505.0 to 722.9)
Non-venomous animal contact	90 178 (73 380 to 110 405)	138 781 (108 637 to 174 760)	500.9 (412.3 to 606.7)	473.2 (374.9 to 589.8)	49 688 (41 614 to 58 461)	78 787 (66 178 to 93 144)	360.8 (305.3 to 421.1)	302.7 (255.2 to 354.5)
Foreign body	60 373 (51 150 to 72 449)	99 397 (84 081 to 117 416)	316.8 (269.6 to 371.1)	335.3 (286.0 to 390.6)	26 822 (21 703 to 32 644)	47 135 (38 208 to 57 057)	168.6 (140.1 to 200.4)	170.4 (140.4 to 204.4)
Pulmonary aspiration and foreign body in airway	1 872 (1 414 to 2 516)	1 975 (1 454 to 2 633)	6.7 (5.2 to 8.8)	6.5 (4.9 to 8.6)	3 806 (2 794 to 4 973)	5 521 (4 228 to 7 005)	21.3 (15.9 to 27.5)	19.1 (14.7 to 24.1)
Foreign body in eyes	50 976 (41 711 to 62 689)	86 455 (71 331 to 104 315)	274.2 (227.8 to 329.0)	291.4 (241.5 to 347.3)	7 930 (3 509 to 12 996)	13 943 (6 495 to 22 494)	46.6 (22.8 to 72.6)	49.2 (24.0 to 76.5)
Foreign body in other body part	7 525 (5 886 to 9 831)	10 968 (8 732 to 13 837)	35.9 (29.0 to 44.6)	37.3 (29.8 to 46.6)	15 086 (12 556 to 18 263)	27 671 (22 980 to 33 765)	100.6 (85.5 to 119.0)	102.1 (86.0 to 123.0)
Environmental heat and cold exposure	17 120 (13 847 to 21 046)	30 695 (25 215 to 37 285)	94.7 (79.4 to 111.5)	107.5 (89.8 to 128.2)	52 615 (44 474 to 61 597)	107 234 (91 533 to 124 983)	371.9 (320.8 to 430.4)	410.9 (353.6 to 478.3)
Exposure to forces of nature	3 065 (2 525 to 3 797)	14 504 (12 135 to 17 647)	14.8 (12.6 to 17.7)	46.6 (39.6 to 55.6)	42 580 (31 031 to 57 168)	347 151 (245 728 to 488 004)	228.1 (167.6 to 303.5)	1 177.3 (843.6 to 1 637.5)
Other unintentional injuries	117 637 (102 923 to 134 415)	195 409 (166 820 to 229 264)	698.2 (619.5 to 779.3)	680.0 (586.2 to 784.8)	231 095 (198 707 to 269 624)	427 750 (363 903 to 503 699)	1 756.7 (1 513.6 to 2 028.3)	1 674.6 (1 427.7 to 1 944.9)
**Self-harm and interpersonal violence**	**36 113 (32 438 to 40 617)**	**58 202 (51 129 to 66 494)**	**190.4 (171.5 to 211.5)**	**192.1 (168.9 to 219.3)**	**561 327 (491 402 to 641 904)**	**1 254 931 (1 098 629 to 1 436 116)**	**3 296.9 (2 916.9 to 3 745.6)**	**4 222.1 (3 725.2 to 4 783.9)**
Self-harm	5 307 (4 585 to 6 079)	8 376 (7 132 to 9 949)	30.6 (26.8 to 34.5)	27.4 (23.8 to 32.0)	11 077 (9 206 to 13 478)	18 078 (15 151 to 21 927)	83.1 (69.5 to 100.1)	70.7 (59.5 to 85.2)
Self-harm by firearm	137 (87 to 199)	218 (139 to 325)	1.1 (0.7 to 1.6)	0.9 (0.6 to 1.3)	308 (270 to 358)	502 (438 to 583)	2.9 (2.6 to 3.4)	2.2 (2.0 to 2.6)
Self-harm by other specified means	5 170 (4 456 to 5 951)	8 158 (6 898 to 9 750)	29.5 (25.8 to 33.4)	26.5 (22.8 to 31.2)	10 769 (8 892 to 13 157)	17 576 (14 664 to 21 392)	80.2 (66.6 to 97.4)	68.4 (57.3 to 82.9)
Interpersonal violence	29 258 (25 702 to 33 768)	47 886 (40 733 to 56 119)	152.0 (133.8 to 173.4)	158.4 (135.3 to 185.0)	549 796 (480 244 to 630 368)	1 113 432 (969 211 to 1 285 774)	3 211.3 (2 830.0 to 3 659.3)	3 714.0 (3 251.1 to 4 231.1)
Assault by firearm	262 (194 to 352)	437 (316 to 613)	1.4 (1.1 to 1.8)	1.5 (1.1 to 2.1)	755 (651 to 876)	1 473 (1 285 to 1 703)	5.5 (4.8 to 6.3)	5.7 (5.0 to 6.5)
Assault by sharp object	3 490 (2 587 to 4 636)	5 807 (4 185 to 7 971)	16.1 (12.3 to 21.1)	18.4 (13.4 to 24.9)	9 443 (7 812 to 11 808)	18 693 (15 513 to 23 440)	62.7 (52.1 to 78.2)	68.7 (57.5 to 85.6)
Sexual violence	-	-	-	-	495 726 (424 827 to 575 758)	1 011 378 (863 453 to 1 177 276)	2 831.0 (2 448.3 to 3 270.4)	3 327.8 (2 855.1 to 3 842.1)
Assault by other means	25 506 (22 379 to 29 516)	41 642 (35 500 to 49 052)	134.5 (118.2 to 154.5)	138.5 (118.3 to 162.3)	43 872 (37 936 to 50 738)	81 888 (70 805 to 95 299)	312.1 (271.1 to 361.1)	311.8 (269.7 to 361.7)
Conflict and terrorism	1 338 (1 139 to 1 638)	0 (0 to 0)	6.7 (5.8 to 8.2)	0.0 (0.0 to 0.0)	240 (187 to 315)	121 548 (81 207 to 178 510)	1.3 (1.0 to 1.7)	431.0 (287.9 to 630.7)
Executions and police conflict	210 (137 to 289)	1 940 (1 566 to 2 599)	1.1 (0.7 to 1.5)	6.2 (5.1 to 8.2)	214 (125 to 331)	1 873 (1 292 to 2 779)	1.2 (0.8 to 1.9)	6.5 (4.5 to 9.5)

Cause	Years lived with disability: All-age counts (95% UI) 1990	Years lived with disability: All-age counts (95% UI) 2017	Years lived with disability: Age-standardised rates per 100,000 (95% UI) 1990	Years lived with disability: Age-standardised rates per 100,000 (95% UI) 2017	Disability adjusted life years: All-age counts (95% UI) 1990	Disability adjusted life years: All-age counts (95% UI) 2017	Disability adjusted life years: Age-standardised rates per 100,000 (95% UI) 1990	Disability adjusted life years: Age-standardised rates per 100,000 (95% UI) 2017
**All injuries**	**89 830 (67 227 to 114 315)**	**182 367 (138 390 to 231 736)**	**610.3 (459.1 to 780.8)**	**674.5 (509.7 to 857.1)**	**881 452 (668 625 to 1 049 545)**	**897 969 (718 350 to 1 092 186)**	**4 522.2 (3 530.3 to 5 286.4)**	**3 173.1 (2 563.6 to 3 849.6)**
**Transport injuries**	**21 315 (15 206 to 28 078)**	**41 749 (29 999 to 55 428)**	**143.6 (103.4 to 188.1)**	**156.3 (112.8 to 206.3)**	**238 976 (165 865 to 308 615)**	**372 304 (240 203 to 546 069)**	**1 322.2 (930.0 to 1 729.2)**	**1 300.4 (842.7 to 1 888.4)**
Road injuries	10 456 (7 617 to 13 771)	23 707 (17 105 to 31 644)	74.1 (54.1 to 97.5)	90.4 (65.4 to 120.4)	211 796 (143 211 to 277 175)	320 393 (197 878 to 478 580)	1 163.5 (796.2 to 1 540.6)	1 120.1 (690.2 to 1 657.6)
Pedestrian road injuries	4 115 (2 943 to 5 534)	8 107 (5 687 to 10 937)	29.0 (20.7 to 39.0)	30.9 (21.9 to 41.8)	112 695 (71 113 to 163 246)	153 358 (72 707 to 285 360)	594.7 (383.8 to 864.1)	550.5 (263.7 to 1 011.5)
Cyclist road injuries	1 527 (1 082 to 2 013)	3 758 (2 637 to 5 027)	10.5 (7.5 to 13.8)	14.1 (9.9 to 18.8)	8 063 (4 653 to 13 527)	23 707 (9 298 to 62 820)	49.1 (29.2 to 81.7)	83.7 (33.0 to 224.7)
Motorcyclist road injuries	3 703 (2 593 to 4 964)	8 968 (6 180 to 12 178)	26.6 (18.9 to 35.8)	34.3 (23.7 to 46.4)	44 061 (26 162 to 68 286)	74 852 (40 811 to 141 069)	255.3 (155.2 to 400.8)	251.0 (134.8 to 474.5)
Motor vehicle road injuries	700 (501 to 929)	1 777 (1 263 to 2 371)	5.0 (3.6 to 6.6)	6.8 (4.8 to 9.1)	44 695 (21 858 to 77 696)	64 599 (27 917 to 135 644)	251.0 (126.7 to 445.1)	220.6 (95.9 to 467.2)
Other road injuries	412 (294 to 558)	1 097 (791 to 1 513)	3.0 (2.2 to 4.1)	4.3 (3.1 to 5.9)	2 282 (1 022 to 4 085)	3 876 (2 061 to 9 231)	13.4 (6.6 to 24.3)	14.4 (7.8 to 33.5)
Other transport injuries	10 859 (7 656 to 14 610)	18 043 (12 873 to 24 279)	69.5 (49.2 to 92.7)	65.9 (47.0 to 88.0)	27 180 (20 031 to 35 815)	51 912 (30 164 to 80 990)	158.6 (119.2 to 207.5)	180.2 (105.5 to 276.9)
**Unintentional injuries**	**59 629 (44 121 to 76 776)**	**117 911 (88 529 to 151 651)**	**412.7 (304.1 to 532.4)**	**440.1 (330.3 to 567.1)**	**510 995 (385 060 to 639 142)**	**358 227 (295 708 to 424 802)**	**2 464.3 (1 951.2 to 2 965.1)**	**1 326.0 (1 097.2 to 1 555.5)**
Falls	17 990 (13 140 to 23 669)	36 708 (26 461 to 49 047)	138.3 (101.5 to 181.3)	147.4 (106.5 to 196.5)	78 983 (57 839 to 101 623)	109 142 (85 684 to 135 751)	547.3 (415.9 to 682.2)	458.3 (363.9 to 559.8)
Drowning	482 (341 to 636)	470 (337 to 624)	3.1 (2.2 to 4.0)	1.8 (1.3 to 2.3)	212 629 (141 281 to 291 059)	53 992 (39 680 to 68 274)	776.3 (531.4 to 1 041.2)	175.9 (129.5 to 220.5)
Fire, heat, and hot substances	12 079 (8 592 to 15 614)	15 810 (11 126 to 20 647)	75.6 (53.9 to 96.8)	56.1 (39.9 to 72.9)	43 265 (30 485 to 56 116)	33 068 (25 257 to 43 321)	225.7 (168.0 to 285.2)	115.5 (88.0 to 149.4)
Poisonings	405 (272 to 572)	863 (576 to 1 243)	2.3 (1.6 to 3.2)	2.9 (2.0 to 4.2)	11 308 (4 754 to 18 846)	5 791 (4 023 to 8 651)	45.4 (21.4 to 71.1)	19.0 (13.3 to 28.2)
Poisoning by carbon monoxide	85 (56 to 122)	178 (117 to 260)	0.5 (0.3 to 0.7)	0.6 (0.4 to 0.9)	1 319 (704 to 1 830)	1 152 (782 to 1 707)	6.8 (3.6 to 9.2)	3.9 (2.6 to 5.6)
Poisoning by other means	320 (209 to 462)	684 (440 to 1 006)	1.8 (1.2 to 2.6)	2.3 (1.5 to 3.4)	9 988 (4 015 to 17 249)	4 640 (3 172 to 6 988)	38.6 (17.5 to 62.9)	15.1 (10.4 to 22.6)
Exposure to mechanical forces	6 399 (4 635 to 8 418)	10 389 (7 381 to 13 934)	43.3 (31.5 to 56.8)	38.7 (27.5 to 51.7)	31 929 (24 463 to 40 709)	24 595 (19 826 to 29 755)	171.3 (134.0 to 215.6)	87.9 (71.2 to 105.7)
Unintentional firearm injuries	511 (360 to 678)	774 (550 to 1 031)	3.2 (2.3 to 4.2)	2.8 (2.0 to 3.7)	2 629 (1 521 to 3 977)	1 709 (1 323 to 2 117)	15.0 (9.1 to 22.7)	6.2 (4.8 to 7.6)
Other exposure to mechanical forces	5 889 (4 259 to 7 830)	9 614 (6 822 to 12 982)	40.1 (29.0 to 53.0)	35.9 (25.5 to 48.4)	29 300 (22 059 to 37 921)	22 886 (18 353 to 27 777)	156.3 (121.9 to 198.9)	81.7 (65.8 to 98.4)
Adverse effects of medical treatment	165 (102 to 248)	317 (196 to 481)	1.0 (0.6 to 1.5)	1.1 (0.7 to 1.7)	18 073 (12 644 to 24 286)	19 078 (14 669 to 23 976)	109.7 (84.9 to 135.2)	75.6 (58.6 to 94.0)
Animal contact	4 389 (2 998 to 5 966)	8 273 (5 578 to 11 273)	29.4 (20.1 to 39.6)	30.0 (20.3 to 40.8)	32 106 (17 595 to 44 139)	29 422 (17 128 to 41 806)	163.0 (92.9 to 217.2)	102.2 (59.4 to 143.6)
Venomous animal contact	3 371 (2 278 to 4 596)	6 837 (4 557 to 9 536)	22.0 (14.9 to 30.0)	24.5 (16.4 to 34.0)	28 960 (15 383 to 39 318)	26 401 (14 732 to 37 883)	146.4 (81.6 to 193.9)	91.2 (51.2 to 130.6)
Non-venomous animal contact	1 018 (690 to 1 461)	1 436 (945 to 2 136)	7.3 (5.0 to 10.6)	5.5 (3.7 to 8.2)	3 146 (1 675 to 7 656)	3 022 (1 926 to 4 150)	16.6 (10.0 to 33.5)	11.0 (7.1 to 15.1)
Foreign body	1 775 (1 226 to 2 438)	2 784 (1 905 to 3 880)	10.6 (7.5 to 14.5)	9.8 (6.8 to 13.7)	17 202 (12 221 to 23 703)	8 860 (7 017 to 11 694)	65.1 (49.4 to 85.9)	31.9 (25.7 to 41.9)
Pulmonary aspiration and foreign body in airway	327 (220 to 456)	406 (265 to 558)	1.7 (1.2 to 2.4)	1.4 (0.9 to 1.9)	13 760 (9 255 to 20 100)	5 800 (4 308 to 8 390)	48.3 (34.7 to 68.4)	21.1 (16.0 to 30.1)
Foreign body in eyes	475 (207 to 839)	842 (390 to 1 456)	2.8 (1.4 to 4.9)	3.0 (1.4 to 5.1)	475 (207 to 839)	842 (390 to 1 456)	2.8 (1.4 to 4.9)	3.0 (1.4 to 5.1)
Foreign body in other body part	973 (702 to 1 261)	1 536 (1 102 to 1 994)	6.0 (4.4 to 7.7)	5.5 (4.0 to 7.1)	2 968 (1 802 to 4 437)	2 218 (1 709 to 2 786)	13.9 (10.0 to 18.9)	7.9 (6.1 to 9.9)
Environmental heat and cold exposure	3 434 (2 438 to 4 506)	5 879 (4 172 to 7 769)	22.8 (16.3 to 29.7)	22.0 (15.7 to 29.1)	7 999 (4 692 to 11 105)	8 649 (5 879 to 11 164)	49.6 (30.0 to 63.7)	33.2 (22.5 to 42.9)
Exposure to forces of nature	2 254 (1 503 to 3 243)	19 509 (13 626 to 27 090)	12.0 (8.1 to 16.9)	65.0 (46.2 to 89.4)	4 078 (3 316 to 5 094)	28 040 (22 167 to 35 645)	20.8 (16.9 to 25.7)	92.6 (73.4 to 117.3)
Other unintentional injuries	10 257 (7 440 to 13 760)	16 908 (11 995 to 23 047)	74.4 (53.7 to 99.7)	65.2 (46.3 to 89.1)	53 423 (37 075 to 66 301)	37 588 (29 255 to 45 781)	289.9 (210.4 to 355.6)	133.9 (104.5 to 162.6)
**Self-harm and interpersonal violence**	**8 886 (6 782 to 11 330)**	**22 707 (17 518 to 28 735)**	**54.1 (41.6 to 67.9)**	**78.2 (60.0 to 97.9)**	**131 480 (91 538 to 168 953)**	**167 438 (125 687 to 203 650)**	**735.7 (528.7 to 945.9)**	**546.8 (409.7 to 664.7)**
Self-harm	1 030 (722 to 1 390)	1 368 (966 to 1 843)	7.2 (5.1 to 9.6)	5.2 (3.7 to 6.9)	106 294 (69 737 to 141 451)	118 578 (86 880 to 146 069)	599.1 (404.7 to 799.7)	386.0 (281.0 to 470.8)
Self-harm by firearm	20 (15 to 26)	29 (21 to 39)	0.2 (0.1 to 0.2)	0.1 (0.1 to 0.2)	9 811 (4 344 to 17 396)	7 912 (4 388 to 13 039)	54.3 (24.6 to 95.2)	25.1 (13.9 to 40.6)
Self-harm by other specified means	1 010 (708 to 1 367)	1 339 (943 to 1 806)	7.0 (5.0 to 9.4)	5.0 (3.6 to 6.8)	96 483 (64 512 to 129 498)	110 666 (81 549 to 135 210)	544.8 (375.8 to 730.9)	361.0 (264.2 to 440.9)
Interpersonal violence	7 822 (5 969 to 10 067)	14 639 (10 981 to 19 228)	46.7 (35.8 to 59.1)	49.7 (37.5 to 64.0)	24 172 (18 668 to 29 465)	41 337 (23 849 to 52 597)	131.8 (102.8 to 161.9)	134.9 (80.0 to 170.1)
Assault by firearm	61 (44 to 80)	108 (78 to 143)	0.4 (0.3 to 0.5)	0.4 (0.3 to 0.5)	1 967 (549 to 3 148)	4 397 (630 to 7 593)	11.0 (3.1 to 17.4)	14.1 (2.2 to 23.9)
Assault by sharp object	451 (327 to 596)	782 (565 to 1 049)	2.8 (2.0 to 3.7)	2.8 (2.0 to 3.7)	2 923 (1 549 to 4 563)	5 782 (1 883 to 8 824)	16.7 (9.3 to 25.9)	19.1 (6.6 to 28.7)
Sexual violence	4 453 (2 999 to 6 507)	9 120 (6 130 to 13 264)	24.8 (16.7 to 36.2)	29.5 (19.9 to 43.0)	4 453 (2 999 to 6 507)	9 120 (6 130 to 13 264)	24.8 (16.7 to 36.2)	29.5 (19.9 to 43.0)
Assault by other means	2 856 (2 040 to 3 781)	4 629 (3 304 to 6 136)	18.8 (13.6 to 24.8)	17.0 (12.2 to 22.6)	14 829 (11 032 to 20 327)	22 038 (12 492 to 30 179)	79.4 (60.2 to 108.9)	72.2 (42.7 to 97.5)
Conflict and terrorism	22 (14 to 31)	6 606 (4 241 to 10 105)	0.1 (0.1 to 0.2)	23.0 (14.9 to 34.9)	910 (891 to 931)	6 606 (4 241 to 10 105)	4.3 (4.2 to 4.4)	23.0 (14.9 to 34.9)
Executions and police conflict	13 (7 to 20)	94 (65 to 136)	0.1 (0.0 to 0.1)	0.3 (0.2 to 0.5)	104 (74 to 124)	917 (866 to 976)	0.5 (0.4 to 0.6)	2.9 (2.8 to 3.1)
